# From Pathways to Targets: Understanding the Mechanisms behind Polyglutamine Disease

**DOI:** 10.1155/2014/701758

**Published:** 2014-09-21

**Authors:** Jonasz Jeremiasz Weber, Anna Sergeevna Sowa, Tina Binder, Jeannette Hübener

**Affiliations:** ^1^Institute of Medical Genetics and Applied Genomics, University of Tübingen, Calwerstraße 7, 72076 Tübingen, Germany; ^2^Rare Disease Center, Calwerstraße 7, 72076 Tübingen, Germany

## Abstract

The history of polyglutamine diseases dates back approximately 20 years to the discovery of a polyglutamine repeat in the androgen receptor of SBMA followed by the identification of similar expansion mutations in Huntington's disease, SCA1, DRPLA, and the other spinocerebellar ataxias. This common molecular feature of polyglutamine diseases suggests shared mechanisms in disease pathology and neurodegeneration of disease specific brain regions. In this review, we discuss the main pathogenic pathways including proteolytic processing, nuclear shuttling and aggregation, mitochondrial dysfunction, and clearance of misfolded polyglutamine proteins and point out possible targets for treatment.

## 1. Introduction

**Figure 1:**
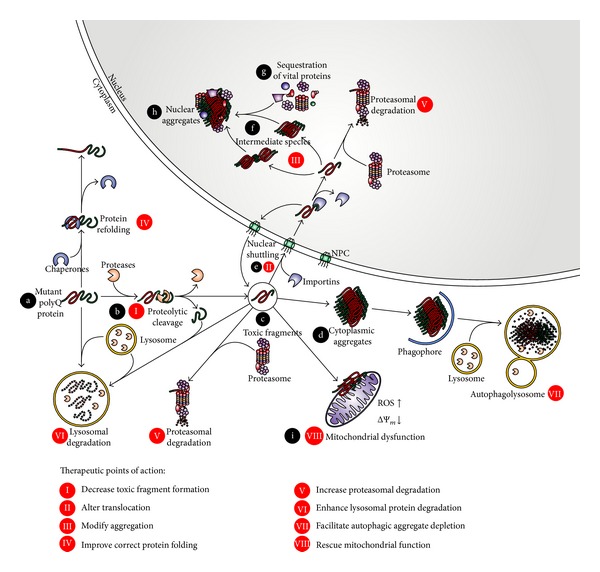
A model of the common molecular mechanisms behind polyglutamine pathology. Schematic illustration of the intracellular fate of the polyglutamine (polyQ) expanded protein, from the unprocessed mutant protein to a protein aggregate. The mutant protein (a) is proteolytically processed by endogenous enzymes (b) forming toxic fragments (c). These fragments form aggregates in the cytoplasm (d). Alternatively, toxic breakdown products can translocate into the nucleus (e) and generate nuclear aggregates (h) by forming intermediate species (f) and sequestering further vital proteins (g). Accumulation of polyQ species can damage important cellular components and lead, for example, to mitochondrial dysfunction (i). The visualized pathways point possible sites for therapeutic engagement: prevention of proteolytic events (I) can decrease levels of toxic fragments. Alteration of nuclear shuttling (II) and modulation of aggregation (III) can ameliorate the detrimental effects of toxic species. As polyQ expansions lead to misfolded proteins, structural refolding assisted by enhanced chaperone activity (IV) might be beneficial. An increased degradation of polyQ proteins and aggregates via proteasomal (V), lysosomal (VI), and autophagosomal (VII) pathways can reduce the amounts of toxic species inside the cell. Finally, attenuating the consequences of polyQ toxicity (VIII), like impaired mitochondrial function, can improve the cellular viability.

Polyglutamine (polyQ) diseases are inherited, fatal neurodegenerative disorders caused by an expansion of a coding trinucleotide (CAG) repeat, which is translated to an abnormally elongated glutamine (Q) tract in the respective mutant proteins. There are nine known polyQ diseases: dentatorubral-pallidoluysian atrophy (DRPLA), Huntington's disease (HD), spinal-bulbar muscular atrophy (SBMA), and six spinocerebellar ataxias (SCA 1, 2, 3, 6, 7, and 17). Except for SBMA, which is X-linked, members of this disease group are inherited in an autosomal dominant manner [1]. It also appears that the shared expansion of polyQ tract confers some shared neurodegenerative pathways on the diseases. Although the region of the brain that is affected differs according to each disease, the observed cell death is aggravated by the trafficking of the protein to specific cellular compartments where it can increase the rate of aggregation. Both nuclear and cytoplasmic aggregates are present in polyQ diseases and contain parts of the respective disease proteins, ubiquitin, and several important homeostatic proteins [2]. The recruitment of ubiquitin, heat shock proteins, and proteasomal subunits into these aggregates implies that protein quality control mechanisms such as the ubiquitin-proteasome system (UPS) are involved in polyQ pathogenesis [3]. It has also been discussed that the cleaved protein is more toxic than the full-length variant. An initial proteolytic cleavage of the respective disease proteins may generate a fragment containing the elongated polyQ stretch which is more aggregate prone and hence more toxic for the cell [4, 5]. What is also interesting about this group of proteins is that although they are all ubiquitously expressed in embryonic stages and adulthood, the pathology of the disease only occurs in neuronal cells [6]. One possible explanation for this phenomenon is the high energy demand of neurons and hence their dependency on oxidative energy metabolism. This points dysfunctional mitochondria as a shared mechanism of neurodegeneration [7]. In this review we focus on what we consider to be the most important pathways in pathology of Huntington's disease and spinocerebellar ataxias: proteolytic processing, nuclear shuttling and aggregation, mitochondrial dysfunction, and intracellular protein degradation systems (Figure 1).

## 2. Proteolytic Processing

Early studies of the common characteristics of polyQ diseases revealed that small fragments of mutant proteins containing the expanded polyQ stretch harbored cytotoxic characteristics [8, 9]. Proteolytic cleavage, the proposed source of these breakdown products, was suggested as an early or initial step in the molecular disease development. This mechanistic concept is commonly known as the* toxic fragment hypothesis *[10]. The presence of proteolytically derived fragments of mutant proteins was reported for all polyQ diseases introduced in this review, namely, SCA 1 [11], SCA 2 [12], SCA 3 [13, 14], SCA 6 [15], SCA 7 [16, 17] SCA 17 [18], and HD [19, 20]. Currently, several classes of endogenous proteases have been linked to the proteolysis of polyQ proteins including the groups of caspases [21–24] and calpains [20, 25–29].

For SCA 1 and 2, neither an inherent cytotoxicity and aggregation propensity nor a clear impact on pathology is evident for mutant protein fragments, demanding further characterization [11, 12]. For ataxin-2, the disease protein in SCA 2, mutant fragment constructs were shown to exhibit an aggregate formation potential* in vitro* [30], but further studies revealed a decreased cytotoxicity of N-terminally truncated mutant ataxin-2 compared to the full-length protein [31]. Even so, for the majority of polyQ diseases a correlation between proteolytic processing of mutant proteins and disease progression is generally accepted.

In a SCA 3 cell model, the expression of a fragment of ataxin-3 containing an elongated polyQ stretch induced apoptosis and cell death as well as a severe ataxia in a mouse model, showing a more rapid manifestation of a SCA 3-reminiscent phenotype when compared to mice expressing full-length mutant ataxin-3 [8]. In addition, polyQ-containing ataxin-3 fragments were shown to form aggregates on their own and were also able to recruit full-length protein into the insoluble inclusions [32, 33]. In HD,* in vitro* data showed that the progressive truncation of mutant huntingtin (mHtt) protein and the length of the polyQ expansion correlate with the aggregation propensity and an increase in apoptotic stress [34, 35]. Mouse studies revealed a similar result when animals expressing the polyQ expanded exon 1 of huntingtin (Htt) showed a progressive neurological phenotype recapitulating characteristics of HD. This suggests that the N-terminal polyQ-containing portion of Htt was sufficient to induce neurodegeneration* in vivo* [9]. An important observation is that these disease fragments were detectable in human HD and SCA 3 brain and lymphoblasts [13, 20, 36] and were found to be an important component of neuronal intranuclear inclusions [37–39]. Similar results were retrieved from two mouse models of SCA 7 expressing polyQ expanded ataxin-7. In brain tissue of these animals N-terminal ataxin-7 fragments were observed which appeared in nuclear aggregates in correlation with onset of the disease phenotype [16, 17]. As with much of the current research on polyQ diseases, not all observations are in agreement. An HD mouse model expressing a polyQ expanded fragment of Htt encompassing exons 1 and 2 exhibited neither neurotoxic effects nor an HD phenotype, despite the presence of nuclear inclusions [40]. This illustrates that not all fragment species feature neuropathological characteristics. Another noteworthy investigation made on a SCA 3 gene trap mouse model showed that expression of a fusion protein comprising *β*-galactosidase and the N-terminal portion of ataxin-3 without the polyglutamine tract led to the formation of cytoplasmic inclusion bodies and to a phenotype reminiscent of the neurological symptoms observed in SCA 3 mice and patients [41]. Furthermore, C-terminal polyQ fragments of the *α*1A calcium channel, disease protein in SCA 6, showed a polyQ independent cytotoxic nature. However, the expansion of the polyQ stretch within the fragment resulted in its increased resistance to proteolysis entailing an accumulation of this toxic species [15].

The first proteases which were shown to cleave polyQ expanded proteins were caspases. This family of cysteine proteases is associated with apoptotic pathways and inflammation but is also known to be involved in a variety of other cellular functions like cell proliferation, differentiation, and migration [42, 43].

Caspases are involved in cell death mechanisms and an increase in activation of caspases has been detected in the course of polyQ diseases. Presence of apoptotic cell death and caspase activation was shown in human HD brains as well as in mouse and cell models of HD [44–51], although this goes against previous studies that did not find apoptotic nuclei in the R6/2 mouse model of HD [52]. Cell death pathways and caspases were also reported to be switched on in other polyQ diseases like SCA 3 [8, 53] and SCA 7 [54, 55]. In the case of SCA 7, activated caspase-3 was recruited into inclusions in cell culture and human SCA 7 brain, and its expression was upregulated in cortical neurons [54]. In general, inhibition of caspases has been shown to ameliorate disease progression and phenotype in HD mice [44, 49].

Within the polyQ diseases reviewed, the first discovery of caspase-mediated cleavage of a disease-causing protein was made for HD [21]. This* in vitro* study indicated a specificity of caspase-3 for huntingtin and a polyQ expansion dependent cleavage. Further studies identified caspase-1 dependent cleavage of huntingtin and confirmed caspase-3-mediated fragmentation, whereas caspases-7 and -8 appeared not to cleave full-length huntingtin [22]. Moreover, caspase-3 selectively processed expanded huntingtin and resulting N-terminal fragments formed cytoplasmic and nuclear inclusions [48]. Direct evidence for caspase-mediated huntingtin cleavage was gained from early stage HD postmortem human tissue and transgenic mice. In these brain tissues, not only mutant but also wild type huntingtin are substrates for caspase cleavage. The early disease stage of these samples suggests that caspase-mediated proteolysis of mHtt may precede neurodegeneration [23].

Multiple studies have begun to elucidate the specific caspases responsible for cleavage of huntingtin. A broad inhibition of caspases with Z-VAD-FMK in clonal striatal cells led to a reduction of specific huntingtin fragments and an increased viability without changing levels of inclusions, whereas treatment with the caspase-3 specific inhibitor Z-DEVD-FMK reduced aggregates without changing cleavage or increasing cell viability [46]. The generation of mouse lines expressing caspase-3 and caspase-6 resistant polyQ expanded huntingtin by eliminating specific cleavage sites unveiled a strong relevance of cleavage at the 586 amino acid caspase-6 site of huntingtin. Removing the caspase-6, but not caspase-3, recognition sites in mHtt appeared to be sufficient to protect from neuronal dysfunction and neurodegeneration* in vivo* [56]. A further study showed that caspase-6, but not caspase-3, is activated before the onset of motor abnormalities in murine and human HD brain. Caspase-6 activation correlated directly with the size of the polyQ expansion and inversely with the age at onset [56]. Moreover, medium spiny neurons (MSNs) expressing caspase-6 resistant mHtt showed a decreased susceptibility for NMDAR-induced excitotoxicity and no caspase-6 activation compared to MSNs expressing unmodified mHtt [56–58]. By contrast, two caspase-6 knockout HD mouse models showed that production of a 586 amino acid derived proteolytic fragment was not prevented in the brain, disagreeing with a direct involvement of caspase-6 in mHtt cleavage [59, 60].

Correlating with the results for huntingtin, caspases-1 and -3, but not caspases-7 and -8, were reported to cleave ataxin-3* in vitro* producing specific fragments [22]. The impact of caspase cleavage was confirmed in a cell based model, showing that predominantly caspase-1-mediated fragmentation of expanded ataxin-3 resulted in increased aggregation and treatment with caspase inhibitors prevented inclusion formation* in vitro* [61]. Interestingly, a different* in vitro* study showed that mutant ataxin-3 was cleaved to a lesser extent than wild-type ataxin-3 after a common initial proteolytic step, suggesting that generated mutant fragments cannot be further degraded. This may result in an accumulation of aggregation-prone expanded ataxin-3 fragment species [62]. In a* Drosophila *model cleavage of ataxin-3 appeared to be conserved and also caspase-mediated, featuring neuronal loss which was mitigated by a sextuplet caspase site mutation in ataxin-3 [63]. A recent publication reported involvement of CDK5 in caspase-mediated ataxin-3 cleavage, showing that RNAi of CDK5 in a* Drosophila* model for SCA 3 resulted in an enhanced SCA 3 toxicity [64]. Contrary to results pointing to an involvement of caspases in the molecular pathology of SCA 3, an* in vitro* study based on patient-derived iPSCs demonstrated that upon excitotoxic stress ataxin-3 cleavage and aggregation were prevented neither by pharmacological inhibition of caspases-1 and -3 nor by treatment with a pan-caspase inhibitor but was abolished by inhibiting calpain activity [65].

In the case of SCA 7,* in vitro* assays identified caspase-7 as the responsible proteolytic enzyme for ataxin-7 fragmentation. The mutation of two specific caspase-cleavage sites in ataxin-7 not only resulted in a resistance of polyQ expanded ataxin-7 to caspase cleavage but also attenuated cell death, aggregate formation, and transcriptional interference in cells. Fragments of ataxin-7 corresponding to products of caspase-7 cleavage were also found in SCA 7 mice, which furthermore exhibited an increased caspase-7 activation and recruitment into the nucleus by expanded ataxin-7 [24]. Nonetheless, full-length expanded ataxin-7 can form inclusions without evidence for cleavage [54].

TBP (TATA-binding protein), the disease protein in SCA 17, was reported to show fragmentation and fragment-dependent formation of aggregates in SCA 17 mice [18], but* in vitro* assays did not show a TBP substrate-specificity for caspases [22], suggesting different proteolytic enzymes to be involved in truncation of TBP.

A second group of proteolytic enzymes that were associated with cleavage of polyQ expanded proteins are calpains, a class of calcium-dependent cysteine proteases. These ubiquitously expressed enzymes exhibit a multitude of regulatory cellular functions and are specialized in modulating structure, localization, and activity of their substrates [66, 67].

In human HD tissue and in brains of HD mouse models an increased expression level of calpains, namely, of calpains-1, -5, -7, and -10, and elevated enzyme activity have been reported [25, 26, 68, 69]. Interestingly, an age-dependent attenuation of calpain activity was observed in an HD mouse model, suggesting alterations in calcium signaling mechanism with disease progression [70]. Furthermore, wild-type and mutant huntingtin were identified as calpain substrates and calpain-dependent proteolytic cleavage products of huntingtin were detected in murine and human HD tissue [25, 27, 46, 71]. Caspase-3 cleavage derived huntingtin fragments undergo further proteolysis by calpains, generating smaller products and suggesting a proteolytic pathway of serial processing events [20]. Additionally, calpain-derived mHtt fragments were shown to accumulate in the nucleus [26], which correlates with cytotoxicity and aggregation in HD [34, 35]. In cell models, the inhibition of calpain cleavage of mutant huntingtin by mutating putative cleavage sites within the huntingtin protein resulted in a decreased proteolysis, aggregation, and toxicity [26]. The mutation of Ser-536 to aspartic acid in order to mimic phosphorylation abolished huntingtin proteolysis at this cleavage site and reduced mutant huntingtin toxicity, pointing to an involvement of phosphorylation events as modulators of calpain cleavage [72]. Concurrent with their activation after ischemic injury, calpains were also shown to cleave full-length huntingtin in infarcted rat cortex and striatum producing N-terminal fragments [73].

Although initial studies stating that an involvement of calpains in SCA 3 was not detectable [61, 63], calpain-dependent proteolysis of ataxin-3 has been reported corresponding to observations in HD [28, 29, 65]. Several putative calpain cleavage sites within the ataxin-3 protein were identified [28, 29, 65, 74], accounting for the generation of a C-terminal polyQ-containing and aggregation-prone fragment [33]. After activation of calpains* in vitro,* fragments of respective sizes were generated. This effect was suppressed when the endogenous calpain inhibitor calpastatin (CAST) was coexpressed in treated cells and aggregation of mutant ataxin-3 was induced or decreased [28]. In a double mutant CAST KO/SCA 3 mouse model, the knockout of the endogenous calpain inhibitor led to higher ataxin-3 fragmentation, amplified aggregate load, increased neurodegeneration, and, in conclusion, to a more severe behavioral phenotype [29]. Reciprocally, overexpression of CAST using adenoassociated viral vectors in a lentiviral mouse model of SCA 3 resulted in reduced ataxin-3 proteolysis and in decreased size and number of intranuclear inclusions of ataxin-3 and neuroprotection via calpain inhibition [74]. In line with these observations, CAST was shown to be depleted in murine and human SCA 3 brain tissue [74]. The neuronal specificity of the molecular mechanisms underlying SCA 3 pathology has been demonstrated by an approach using SCA 3 patient-derived IPSCs. After neuronal differentiation and glutamate-induced calcium influx, excitation-induced ataxin-3 cleavage and aggregation were triggered. This was observed only in neurons, not in glial cells or fibroblasts, and was abolished by calpain inhibition [65].

Although caspases and calpains are reported to account for the majority of cleavage effects on polyQ expanded disease proteins, several fragmentation events could not be explained by their proteolytic activity. An important group of enzymes to consider is the lysosomal cathepsins, which has been shown to process mutant huntingtin. An involvement of cathepsins-D, -B, -L, and -Z [75–77] has been indicated to produce fragments termed cp-A and cp-B [78]. For the cp-A fragment it was illustrated that the protease responsible for its formation has cathepsin-D-like properties in immortalized neurons and gamma-secretase-like properties in primary neurons, pointing to a cell type specific involvement of different proteolytic enzymes [79]. A further screen for identification of novel proteases using 514 protease-specific siRNAs detected 11 enzymes including three members of the matrix metalloproteinase (MMP) family cleaving huntingtin. When knocking down the most promising candidate MMP-10 in a striatal cell line cleavage of mutant huntingtin was prevented. In line with this work, MMPs were shown to be upregulated in HD mouse models and loss of function of* Drosophila* MMP homologs also ameliorated mutant huntingtin-induced neuronal dysfunction [80]. A very interesting novel explanation for the appearance of toxic fragments of huntingtin is that observed missplicing of huntingtin transcripts accounts for shortened N-terminal huntingtin variants [81]. A likewise fascinating attempt to explain ataxin-3 cleavage was done by showing that the intrinsic proteolytic property of ataxin-3's Josephin domain may lead to an autolytic processing of the disease protein [82]. However, C14A ataxin-3 mutants lacking proteolytic activity exhibited no differences neither in subcellular localization nor in proteolysis [62].

As a multitude of publications show that proteolytic processing of polyQ expanded proteins by a variety of enzymes represents a pivotal step in the molecular pathomechanism of polyQ diseases, modulating the activity of cleavage-responsible proteases or decreasing the levels of toxic fragments could be reasonable approaches for therapeutic treatment.

There are various ways to approach treatment. One method is to inhibit the proteolytic activity of caspases, calpains, cathepsins, or MMPs directly. Using such methods beneficial effects were achieved for HD [26, 46, 80, 83, 84] and SCA 3 [28, 61, 63, 65]. But attention should be paid to potential adverse effects as well [84]. A similar approach is to target the expression of endogenous inhibitors, such as calpastatin, as was done in SCA 3 [28, 29, 74].

A second approach is to modulate alternate pathways and achieve off-target benefits. Treating R6/2 mice with a tetracycline derivative delayed disease progression and death by reducing the levels of caspases-1 and -3 [42] through upstream regulation of Apaf-1 [85]. Reducing elevated calpain activity in HD mice also had beneficial off-target benefits [68, 69]. In addition, CDK5 was reported to act against caspase cleavage of huntingtin by phosphorylation at S434 [86]. In SCA 3, decreasing CDK5 levels via RNAi in* Drosophila* enhanced mutant ataxin-3 toxicity [64]. Another option is to use a genetic approach to modulate cleavage such as induction of exon 12 skipping in huntingtin pre-mRNA using oligonucleotides. This modification prevented the translation of the caspase-targeted region around amino acid 586 and thereby inhibited the formation of an N-terminal fragment implicated in HD toxicity [87].

## 3. Aggregation

As the pathological hallmark of polyQ diseases [1], aggregation has been widely discussed as therapeutic target. Although it serves as an easy readout for screens, cell models, and neuropathology, the exact role of aggregates in the neurodegeneration observed in polyQ diseases is still under debate. In the field of polyQ diseases, aggregates were identified as intranuclear inclusions in mouse models of Huntington's disease [52] and subsequently confirmed in HD patients [37, 88]. This was quickly followed by an identification of aggregates containing the disease protein in SCA 3 [32, 38] and SCA 1 [89] and cytoplasmic aggregates in SCA 6 [90], SCA 7 [54, 91], and SCA 17 [92]. In SCA 2, the initial reports of the absence of aggregation [93, 94] have since been challenged [95, 96].

What was initially observed as large fibrillar inclusions is most likely the end stage of protein aggregation and nucleation. The beginning steps feature monomeric species which transform into oligomeric structures and protofibrils/fibrils, although the correlation between these intermediates may not be linear. Some of these species may be direct pathway intermediates while others may not be directly relevant to the inclusion formation seen in patients [97]. Recent advances are being made and assays developed which will help in studying this pathway of aggregation, monomer addition, and isolating specific aggregate species [98]. Work in the field of HD on oligomer formation is bringing the field closer to understanding the mechanisms behind nucleation. The conversion of monomers to oligomers in HD is described as a packing of the N-terminal Htt segment into the oligomer core [98], elongation of fibrils follows, and a third step involves the ability of oligomers to seed monomer elongation. The work suggests that oligomer dissociation rates are similar to association rates and that oligomers serve as both on-pathway and off-pathway intermediates in fibril formation. It seems important to thus consider the aggregation pathway as an ebb and flow of intermediates which feed into multiple pathways. Ataxin-3 was also shown to have a multistep aggregation process where the first step involves the aggregation of the protein independent of the polyQ domain and a second step which is unique to the polyQ expansion and produces highly stable amyloid-like aggregates [99]. In a discussion about aggregation pathways, it is also important to note that kinetic differences between nucleation and protein folding in the nucleus and in the cytoplasm probably play a large role in the observed differences we see in inclusions between nuclear protein aggregates such as in SCA 1 and SCA 3 and cytoplasmic proteins such as SCA 2 and SCA 6 [100]. For HD, the study of the aggregation pathway pointed to “at least three” aggregation pathways which can be influenced by various inhibitors, molecules, and interactions [101]. Inhibiting each pathway has different effects on neurotoxicity. The same was shown for ataxin-3 where different amyloid aggregates affect Ca^2+^ regulation by different mechanisms [102]. Altering the specific pathways of aggregation is a potential therapeutic strategy which may not decrease the total amount of aggregation but could decrease neurotoxicity.

A widely discussed topic is the exact cytotoxic nature of aggregates. By looking at the specific location of inclusions in patient brains, a discrepancy arose between the neurons which have the inclusions and the neurons which are known to degenerate [103]. In HD, the medium spiny neurons which are selectively lost present with much less aggregation than the large interneurons [103]. This and similar findings suggest that the large aggregates are protective. But the other work, such as in SCA 1, reiterates the relationship between aggregates and cytotoxicity. Patients who have a specific histidine interruption in the expanded polyQ tract of ataxin-1 have a decreased amount of aggregation and absence of disease [104]. The issue with such findings is that it does not provide insight into what is happening with intermediate oligomeric species which are more correlated to the onset of symptoms than to the formation of large protein aggregates [105, 106].

Just to highlight how complex it is to tease out the exact role of aggregates, in SCA 7 and SCA 6, different types of nuclear inclusions were identified. In SCA 7, they differ in their size, composition, and distribution of key proteins [54, 91] and detection with a p62 antibody found different subsets of cytoplasmic aggregates in SCA 6 [107]. Furthering our understanding of the interplay between neuronal types can tell us more about the effect of aggregation in specific populations and how that affects the health of surrounding cells. It is difficult to come to a conclusive decision on aggregation and to pull apart the protective properties from the cytotoxic ones without further information.

The discussion on toxicity of aggregates is also relevant for the screening of large libraries of therapeutic compounds or genetic modifiers. Using aggregation as readout is intuitive since if any part of the pathways of aggregation is toxic, then reducing the eventual product of large readout aggregates could also be considered reducing the intermediate toxic species. However, the field should be cautious about blocking the conversion of toxic oligomeric species to possible beneficial aggregates or shifting the balance of different conformation in an unfavorable direction [108]. It is also possible to look at increasing the overall rate of aggregation which could decrease the amount of time in which toxic intermediates can do damage but would cause an overall increase in total large aggregates. Targeting the depletion of specific species with antibodies or upregulated clearance is also a therapeutic possibility. Also, although extracellular aggregate transmission has not been proven for polyglutamine diseases, it could be possible to target the prion-like spread of smaller fibrils and oligomers [109, 110].

In general, a focus on aggregation has allowed the field to gain knowledge about various biological pathways involved in polyQ induced neurodegeneration. As previously described, cleavage plays a large role in the kinetics of aggregation and the mechanisms of toxicity. In the search for intermediate steps between proteolysis and aggregation, it was demonstrated in various cell models that polyQ-containing fragments or polyQ stretches themselves are generally able to form soluble oligomeric structures, mediating cytotoxicity and representing a starting point for subsequent aggregation [111–113]. These oligomeric species could also be identified in brain tissue of HD mouse models and patients [113, 114].

Looking at aggregates has also allowed us to see the recruitment of proteasomal subunits and look into the dysregulation of the ubiquitin system in neurodegeneration. But aggregation is slowly becoming an avoided topic in polyQ research. Hopefully recent advances in understanding aggregate intermediates will open a door to a better analysis of aggregation in neurodegeneration. This can lead to a renewed interest in understanding the complexity behind protein folding and nucleation in polyQ diseases.

## 4. Nuclear Transport

One aspect relevant to both of the aggregation of these proteins and to their general function is their ability to shuttle between the nucleus and cytoplasm. This transport modulates how they both perform their regular function and cause neurodegeneration. Nuclear transport encompasses many features of protein function such as transcription, avoidance of protein clearance machinery, import of a toxic fragment, and many other cellular processes. The current evidence suggests that the nucleus is a large site of toxicity in cells and blocking nuclear transport in animal models has shown that this pathway is a possible therapeutic target [109, 115, 116]. In general, nuclear entry is a highly controlled process and at the heart of that regulation is the nuclear pore complex which serves as a selective gatekeeper of entry [117]. The nuclear pore complex recognizes a group of proteins known as karyopherins which are carrying protein cargo for entry and exit out of the nucleus. Karyopherins recognize their cargo by the presence of specific nuclear localization signal (NLS) and nuclear export signal (NES) on proteins (reviewed by [118]). The most direct way for a protein to be transported by a karyopherin is to have an identifiable NLS or NES (or combination), but secondary features such as the visibility of this signal and posttranslational modifications such as phosphorylation and cleavage which alter the signal also play a large role.

Within the polyQ diseases discussed here, NES and/or NLS have been found for the disease proteins of SCA 1, SCA 3, SCA 7, and HD [11, 119–123].

In SCA 1, specifically, it has been shown that regulation of nuclear localization is relevant to disease progression and ataxin-1 stability. It was shown early on that blocking the NLS on ataxin-1 prevents the protein from causing neurodegeneration* in vivo* [11]. It was later explained that phosphorylation at S776 and the subsequent binding and release from 14-3-3 can mask the NLS, stabilize ataxin-1, and modulate its localization [124] which is important for the nuclear interaction of ataxin-1 with splicing factors RBM17 and U2AF65 [125]. 14-3-3 is a protein that is involved in regulating many cellular processes by binding phosphorylation sites and the example of ataxin-1 demonstrates how factors outside of direct nuclear shuttling influence localization and affect the direct pathomechanisms of disease.

SCA 3 and HD have been the two most widely studied in the possible therapeutic regulation of transport to modify disease. The focus on nuclear transport has been a consequence of studies where fusing mutant fragments of Htt to exogenous NES prevented nuclear transport and inhibited the toxicity of the fragment [126, 127] and the reverse happened when it was fused to an NLS [126]. This work was reproduced in a mouse model which had a shorter lifespan correlated to an added NLS [128]. It has been difficult to tie together the cellular events that cause transport with the known pathways of nuclear entry. It is known, for example, that ataxin-3 and huntingtin enter the nucleus in response to cellular stress and heat shock, but the exact mechanism of transport is not elucidated [129–131]. Also, phosphorylation of both proteins has been linked to nuclear transport. Phosphorylation of huntingtin on N17 releases it from the endoplasmic reticulum to allow nuclear entry but also prevents export from the nucleus during stress response [132] and modulates its neurotoxicity [133]. In the case of ataxin-3, CK-2 dependent phosphorylation of S340 and S352 within the third UIM (ubiquitin interacting motif) has been suggested to control nuclear entry [134]. The current research is also focused on understanding the karyopherins involved in the recognition of the NLS and NES sites of these proteins with the aim of modulating disease. CRM1, or exportin-1, has been shown to interact with both ataxin-3 and huntingtin NES sites [132, 135] and suggested to be an exporter of ataxin-7 [122]. Karyopherins B1 and B2 have also been published as possible mediators of huntingtin localization which act on a putative huntingtin NLS [136]. Cellular and oxidative stress were shown to alter the activity of CRM1 and to affect the localization of polyQ proteins by posttranslational modifications of karyopherins or subsets of the nuclear pore complex [137].

Also of note is the importance of the NLS site in SBMA. The androgen receptor (AR) is kept in the cytoplasm by heat shock proteins which mask this nuclear localization site but, upon binding to the androgen ligand, the NLS is exposed and the androgen receptor translocates to the nucleus where it activates androgen-responsive genes (reviewed in [138]). The presence of the androgen receptor in the nucleus in the presence of the ligand is considered necessary for disease development as mice with an NLS deletion showed delayed onset of phenotype and reduced motor deficit [139]. It is important to note this nuclear function of the AR as the proteins in SCA 1, SCA 3, and HD may also have similar important roles in the nucleus, although aggravating their nuclear presence may overwhelm those beneficial roles and cause neurotoxicity.

In those polyQ diseases where an NLS or NES has not been identified, localization of the protein has still proved to be important to pathogenesis. Recent work using a polyQ antibody has demonstrated that the localization of ataxin-2 within the cell corresponds to disease stages of SCA 2. Cytoplasmic presence corresponded to early stage and nuclear presence and aggregation to final stages of the disease [140]. The mislocalization of ataxin-2 has also been shown to be a potent modifier of ALS/TDP43 toxicity [141] and it has also been suggested that ataxin-2 is important for SCA 3 neurodegeneration. This points to the possibility that the localization of ataxin-2 is important in modulating other neurodegenerative diseases [142]. In SCA 6, the C-terminal peptide of the alpha 1A subunit of the P/Q-type voltage-gated calcium channel with the expanded polyQ tract is also toxic to cells depending on its nuclear localization [143]. Although the exact mechanism behind this translocation is not known, the current hypothesis is that it is important for disease progression.

One way to affect localization is to target the polyQ expansion of the protein. It was shown that the expansion of the CAG repeat in Htt reduces its interaction with Tpr, a nuclear pore protein, which is involved in nuclear export [144] and the expansion of ataxins-3 and -7 has also been linked to nuclear retention [122, 145].

Overall, nuclear trafficking and localization are a summation of many processes that happen within the cell starting from cleavage of the protein, aggregation, modulation of mitochondrial response, and involving all functions of the protein such as transcriptional regulation. The list of proteins with altered subcellular localization in neurodegeneration includes NFkB, ERK1/2, TDP43, Smad, E2F1, CREB, and many others [146]. Because of this wide breadth of cellular mechanisms involved in nuclear localization, it should always be considered an aspect of therapeutic intervention.

## 5. Clearance Mechanisms

It is known that polyQ proteins are associated with the formation of intracellular aggregates, possibly through the formation of toxic fragments, but the important question of what clearance mechanisms are involved remains. The two main clearance routes of organelles and proteins in eukaryotic cells are the ubiquitin-proteasome system (UPS) together with heat shock response and the autophagy-lysosomal pathway. While proteasomes predominantly degrade short-lived nuclear and cytoplasmic proteins as well as misfolded and unfolded proteins from the endoplasmic reticulum, the autophagic system can degrade organelles and cytoplasmic protein complexes [147, 148].

The interplay of heat shock proteins, chaperones, and the UPS is important for protein clearance [149]. During oxidative or cellular stress heat shock proteins are dramatically upregulated. They bind to misfolded proteins and remodel them back to their native formation. If refolding is not possible, degradation by the proteasome is initiated. Failure in one of the systems can be compensated partially by the upregulation of the other, but prolonged failure results in protein aggregation and dysfunctional homeostasis of cells [150]. Many wild-type ataxins as well as huntingtin have been shown to interact with components of the UPS under normal conditions. Yeast two hybrid assays demonstrated an interaction of ataxin-3 and the ubiquitin and proteasome binding factors HHR23A and HHR23B [151, 152]. Ataxin-1 was shown to interact with the ubiquitin-like protein A1Up [153], the ubiquitin-specific protease USP7 [154], and the E2 ubiquitin-conjugation enzyme UbcH6 [155, 156]. Moreover, ataxin-7 was indicated to interact with the S4 subunit of the 19S proteasome [157].

In line with the fact that normal function of polyQ proteins involves interaction with the quality control system is the knowledge that molecular heat shock proteins, ubiquitin, and proteasomal subunits are found in neuronal aggregates in postmortem brains of patients. In HD patients and animal models, aside from the N-terminal part of mHtt, ubiquitin, molecular chaperones including GRP78/BiP, HSP70, and HSP40, and the 20S, 19S, and 11S subunits of the 26S proteasome were also found ([37, 158], reviewed in [159]). Similar results were described for SCA 1 [160], for SCA 3 [161], and for SCA 7 [54, 157]. Together, these findings indicate that ubiquitin, heat shock proteins, and subcomplexes of the 26S proteasome are redistributed to the site of polyQ protein degradation.

The carboxyl terminus of the HSC70-interacting protein (CHIP) is a HSP70 cochaperone as well as an E3 ubiquitin ligase that protects cells from proteotoxic stress. The ability of CHIP to interact with HSP70 and function as a ubiquitin ligase places CHIP in a pivotal position in protein quality control [162] and makes CHIP a frequently analyzed protein in polyQ refolding and degradation. It was shown that CHIP directly interacts and colocalizes to ataxin-1, ataxin-3, and huntingtin aggregates [163, 164]. Additionally, CHIP promotes ubiquitination of wild-type and mutant ataxins-1 and -3 and huntingtin as well as decreasing steady state levels of mutant ataxins-1 and -3 and huntingtin by inducing degradation. Therefore, CHIP suppresses aggregation and toxicity in cell culture and* Drosophila* [163, 164]. Suppression of CHIP resulted in an increased formation of microaggregates and toxicity in a SCA 3 transgenic mouse model [165]. Moreover, overexpression of CHIP together with ataxin-1 led to reduction of ataxin-1 solubility and thus increased formation of aggregates [166]. Another HSP70-dependent E3 ligase that is shown to act redundantly to CHIP on some substrates is parkin [167]. Parkin (*PARK2*, mutated in an autosomal recessive form of PD), which mediates the targeting of proteins for proteasomal degradation, is known to interact and modulate ataxin-2 and ataxin-3 but not ataxin-1 [166, 168–171]. Wild-type and polyQ expanded ataxin-3 deubiquitinate parkin directly and parkin ubiquitinates and facilitates the clearance of wild-type and mutant ataxin-2 and ataxin-3 by proteasomal degradation [168–170]. Additionally, it was demonstrated that parkin forms a complex with the expanded polyQ protein, HSP70, and the proteasome. This decreases cytotoxicity in SCA 2 and SCA 3 by reducing proteasomal impairment. No direct interaction of huntingtin and parkin has been described to date although studies confirmed the colocalization of parkin and huntingtin in mouse brain as well as in patient samples [168]. Additionally, a partial suppression of parkin in an HD mouse model slightly aggravates the neurological phenotype [172]. The interaction or modulation of polyQ disease proteins by parkin can offer an explanation of the parkinsonian phenotype in SCA 2 and SCA 3. Also it is noteworthy that ataxin-1 interacts and is modulated by an E2 ubiquitin-conjugation enzyme, called UbcH6, which regulates the transcriptional repression of expanded ataxin-1 and the rate of ataxin-1 degradation [155, 156]. The binding and ubiquitination of huntingtin by the E2 ubiquitin-conjugation enzyme E2-25K is not influenced by the length of the polyQ stretch [173]. But it is shown that the expression of E2-25K modulates the aggregation and toxicity of mutant huntingtin and that E2-25k is recruited to aggregates in HD and SCA 3 patients [174]. Together these findings indicate a clear influence and impairment of the UPS in all polyQ diseases discussed with the exception of SCA 6. Here, the proteasome has not been implicated in disease progression and there is no evidence for the ubiquitination of aggregates.

Unfolding and remodeling of proteins is necessary for them to pass through the narrow pore of the proteasome barrel, which thus precludes clearance of oligomers and aggregated proteins [175]. A number of polyQ diseases have been associated with decreased chaperone and proteasome activity in patients, cell, and animal models of SCA 1, SCA 3, SCA 7, SCA 17, and HD [176–182]. Nonetheless, there was work demonstrating that, in a SCA 7 knock-in mouse model, no significant impairment of the UPS was found [183]. Also, in recent studies on HD degradation, rapid and complete clearance of polyQ expanded huntingtin in neuronal cells and* in vitro* was shown [184] and dynamic and reversible recruitment of proteasomal subunits into inclusion bodies was observed in living cells [185]. In addition, several groups demonstrated that inhibition of the proteasome in cell culture and mammalian cells results in increased aggregation and cytotoxicity in SCA 3 and HD [181, 186], whereas an overexpression of p45 (ATPase of 19S subunit of proteasome) stimulates degradation of ataxin-3 [187]. Whether the proteasomal enzymatic machinery is able to cleave between successive glutamine residues remains unclear [184, 185, 188–190].

One widely accepted theory is that degradation of misfolded polyQ proteins is a team effort between autophagy and the UPS. Besides the above mentioned involvement of the UPS it is known that the aggregation-prone polyQ proteins and fragments strongly depend on autophagy for their clearance [191]. In SCA 7, the unmodified truncated protein was shown to be degraded via macroautophagy* in vitro *[192] and it was shown that macroautophagy and proteasomal degradation play a role in degrading mHtt [76, 184]. In these studies they demonstrated that blocking autophagy resulted in reduced cell viability and increased number of aggregates and stimulating autophagy promoted clearance of wild-type and mutant huntingtin as well as its caspase derived N-terminal fragment of huntingtin [76]. Specifically targeting the N-terminal huntingtin for the UPS decreased its levels and thus decreased aggregation [184]. Furthermore, it was shown that a polymorphism in an autophagy related gene (ATG7) modulates the age at onset of HD patients [193, 194].

For SCA 1, SCA 3, SCA 6, and SCA 7 an increased susceptibility of cytoplasmic aggregates to autophagic degradation was shown compared to nuclear polyQ inclusions [195–200]. Impairment of the autophagic system is demonstrated by an increased number of autophagosomes, endosomal-lysosomal-like organelles, and multiple vesicular bodies. This was shown in brain and lymphoblasts of HD patients and in primary neurons and brain of HD transgenic mice [52, 201–203]. Characterization of a SCA 1 transgenic mouse model also indicated changes in the autophagic flux by vacuolar formation with autophagic origin and significant altered LC3-II/-I ratio [204]. Similar results were found in ataxin-7 transgenic mice where LC3 levels were significantly altered and wild-type ataxin-7 levels were stabilized by autophagy whereas no stabilizing effects were described for mutant ataxin-7 [196]. Additionally, it was shown that full-length and cleaved fragments of ataxin-7 are differentially degraded. While full-length wild-type and mutant ataxin-7 was primarily found in the nucleus and therefore degraded by the UPS, fragments of ataxin-7 which were located in both the cytoplasm and nucleus were found to be degraded similarly by autophagy and the UPS [197]. Pharmacological activation of autophagy by treatment with a p53 inhibitor led to increased autophagic activity together with reduced ataxin-7 toxicity and therefore represents a possible therapeutic approach in the treatment of SCA 7 [205].

p62 acts as a cargo receptor for degradation of ubiquitinated targets by autophagy [206]. Studies in human postmortem brain samples from SCA 3, SCA 6, and HD patients revealed p62 positive cytoplasmic, axonal, and nuclear aggregates. This again indicates an involvement of the autophagic system in the clearance of aggregated polyQ proteins [107, 207, 208]. p62 also contributes to recruitment of proteasomes to nuclear aggregates of ataxin-1 and to the degradation of ataxin-1 [209]. As discussed earlier, mammalian proteasomes may not be able to cleave (polyQ) sequences and seem to release polyQ-rich peptides. An initial study about a cytosolic enzyme called puromycin-sensitive aminopeptidase (PSA) showed that it is able to digest polyQ sequences [210]. However, in cultured cells,* Drosophila*, and mouse muscles, PSA overexpression decreased aggregate content and toxicity of mutant huntingtin and mutant ataxin-3 by enhancing autophagy [211].

As discussed earlier in this review, aggregates including polyQ protein fragments are believed to cause neuronal death. Therefore, reducing the amount of aggregates is an important therapeutic strategy. This reduction can be achieved by enhancing the above described mechanisms: chaperone mediated refolding of polyQ proteins or degradation of misfolded proteins by autophagy or the UPS. Heat shock proteins were shown to accumulate in aggregates of HD, SCA 1, SCA 3, and SCA 7 and this led to an interest in modulating the molecular chaperone machinery as a possible therapeutic strategy for polyQ diseases. An overexpression of HSP40/HDJ-2 suppressed ataxin-3 and ataxin-1 aggregation* in vitro* [3, 160], but not in huntingtin exon 1 overexpressing cell lines [185]. Moreover, modulation of the chaperone system in HD, SCA 1, SCA 3, and SCA 17 studied* in vitro* [212, 213], yeast [214],* C. elegans* [215],* Drosophila* [216], mammalian cells [186, 217–219], and animal models [220–226] demonstrated controversial results. As the overexpression of single members or the combination of different members of the molecular chaperone system gave controversial and transient effects, the development of combinatorial therapies was proposed. Combining treatment with histone deacetylase (HDAC) inhibitors was promoted in recent years. It was shown that the oral administration of 17-(allylamino)-17-demethoxygeldanamycin (17-AAG) markedly suppressed eye degeneration, inclusion formation, and lethality in a SCA 3* Drosophila* model and also neurodegeneration in an HD* Drosophila* model by induction of HSP70, HSP40, and HSP90 expression [227]. Valproic acid (VPA) an antiepileptic drug which also acts as an HDAC inhibitor and promotes expression of small molecules including HSP70 was shown to alleviate the phenotype of SCA 3 in* Drosophila* [228] and in HD transgenic mice [229]. Furthermore, a combined treatment of lithium (induces autophagy and downregulates HDAC1) and VPA produced several beneficial effects and prolonged median survival in HD transgenic mice [230]. In HD patients, valproic acid is discussed to have beneficial effects on psychiatric symptoms [231] but was also shown to have side effects like developing Parkinson's syndrome with an axial dystonia [232]. The HDAC inhibitor sodium butyrate was shown to delay the onset, ameliorate the neurological phenotype, improve the survival in SCA 3 transgenic mice, and improve the survival of neurons in an ataxin-7 cell model [55, 233]. An analog of this compound, sodium phenylbutyrate, was successfully tested in HD mice [234] and was shown to be safe and well tolerated by HD patients [235], but a phase II clinical trial (started 2006) was abandoned with no cited results.

Although attempts at modulating the proteasome system have been made, upregulation of this pathway is challenging and thus attention has shifted to enhancing autophagy [236]. In polyQ diseases, it has been demonstrated that modulation of one system has direct effects on the other. An HSP90 inhibitor (17-DMAG) resulted in a reduction of neuropathology in a SCA 3 transgenic mouse model although the biggest induction was of LC3-II and beclin and not in heat shock proteins as expected [237]. Beclin modulation has been previously shown to rescue motor symptoms and ataxin-3 clearance in a lentiviral-based rat model [199, 200] and in HD cell culture and primary neurons [237, 238].

Autophagy can also be upregulated by mTOR- (mammalian target of rapamycin-) dependent and mTOR-independent pathways. Autophagy can be induced in all mammalian cell types by rapamycin, an inhibitor of mTOR. Rapamycin treatment of cells expressing aggregation-prone polyQ disease proteins enhanced the degradation of polyQ proteins, reduced the number of aggregates, and protected cells, flies, and mice from mutant protein-associated degradation in SCA 3 and HD [239–241]. Lithium, which is normally used to treat bipolar disorders, was shown to have beneficial effects in polyQ diseases by an mTOR-independent pathway. It targets various intracellular enzymes, including glycogen synthase kinase 3*β* and inositol monophosphatase by lowering inositol and IP3 levels [242]. Induction of autophagy by lithium led to enhanced clearance of autophagy substrates, like mutant huntingtin fragments as well as mutant ataxin-1 and ataxin-3* in vitro*, in* Drosophila* and mouse models [240, 243–246]. Additionally, a combinatory treatment of lithium and rapamycin protected an HD* Drosophila* model against neurodegeneration by enhancing macroautophagy [247]. Other substances having a beneficial effect on mutant huntingtin toxicity and clearance by activating an mTOR-independent pathway are rilmenidine and trehalose [248]. Trehalose together with rapamycin again showed an additive effect on the clearance of mutant huntingtin [249]. Very recently, the first nanomedical approach in treating HD was presented. It was demonstrated that europium hydroxide nanorods reduced huntingtin aggregation by inducing autophagic flux [250].

## 6. Mitochondrial Dysfunction

As the field of research in polyQ diseases is progressing, more is understood about the common mechanisms behind neurodegeneration. Over the last decade an emerging role in the pathogenesis of several neurodegenerative disorders such as Alzheimer's disease (AD), Parkinson's disease (PD), and amyotrophic lateral sclerosis (ALS) [251, 252] has been assigned to mitochondrial dysfunction and impaired energy metabolism. This can be explained by the high energy demands of neuronal cells and their inability to produce ATP by glycolysis and hence dependence on functional mitochondria for oxidative phosphorylation. Recent findings also support the involvement of dysfunctional mitochondria in polyglutamine diseases. Most insights were gained in the field of Huntington's disease but several studies also highlight the role of mitochondria in the pathology of spinocerebellar ataxias.

Metabolic defects and loss of body weight at early stages of the disease are well described symptoms of polyQ disease patients in HD [253, 254], SCA 1 [255], and SCA 3 [256] as well as in the respective disease mouse models [9, 41, 257]. For HD and SCA 3 patients, an inverse correlation between body mass index and CAG repeat number was reported [256, 258]. In SCA 1 patients this weight loss appears despite a balance between energy intake and expenditure and patients show an increase of energy expenditure and fat oxidation at a resting state which might be a cause of altered autonomic nervous system activity and gait ataxia [255].

Another common feature of polyQ diseases is metabolic alterations. Advanced magnetic resonance imaging techniques are used to study alterations in metabolite concentrations in distinct brain regions of patients and mouse models. Increased lactate production was found in cortex and basal ganglia of HD patients [259] while cerebellum and brain stem of SCA 1 patients showed decreased total NAA (N-acetylaspartate + N-acetylglutamate, tNAA) concentrations and elevated glutamine, total creatine, and myoinositol concentrations compared to controls [260, 261]. The levels of tNAA and myoinositol correlated with patients' ataxia scores. Similar changes in metabolite concentrations were seen in conditional SCA 1 and a SCA 1 knock-in mouse models. Interestingly, the metabolite levels almost went back to baseline when expression of the transgene was suppressed at early stages of the disease in the conditional mouse model and alterations in metabolite levels were observed in knock-in mice months before any pathology was detected [261, 262].

Apart from alterations in metabolite concentrations, oxidative stress and changes in ATP production caused by deranged respiratory chain complex activities indicate mitochondrial dysfunction in polyQ disease. As previously reviewed, HD patients show reduced complexes II, III, and IV activities in putamen and caudate, while alterations in complex I activity were found in muscles only [263]. Several studies also point to dysfunctional respiratory chain complex and increased oxidative stress in SCA 2, 3, and 12 [264–270]. Decreased complex II activity was found in lymphoblasts from SCA 3 patients, in cells from transgenic mice and in SCA 3 cell models [269]. In cells expressing human, polyQ expanded ataxin-3, decreased activities of the antioxidant enzymes catalase, glutathione reductase and superoxide dismutase, and consequently mitochondrial DNA damage were detected [266]. Similar findings of increased catalase levels and DNA damage were gained from SCA 3 patient samples compared to healthy controls [270]. A recent study also suggests that the disease characteristic aggregates can be reduced in a neuronal SCA 3 cell model by treatment with an extract of* Gardenia jasminoides* which was shown to reduce the production of reactive oxygen species [271].

While the precise pathways which lead to the observed problems in mitochondrial bioenergetics remain elusive, localization of polyQ disease causing proteins to the mitochondria and their actions at the mitochondria have been subjects of intensive research. For SCA 3, it is known that both normal and polyQ expanded ataxin-3 localize to mitochondria [62] and that degradation of polyQ expanded ataxin-3 via the UPS is promoted by an ubiquitin ligase in the outer mitochondrial membrane called MITOL [272]. Localization to the mitochondria was also shown for mutant huntingtin. Also, mitochondria from HD patient lymphoblasts and from brain of transgenic mice expressing full-length mHtt had decreased membrane potential and defects in mitochondrial calcium handling [273].

An important role in regulating mitochondria mediated cell death in polyQ disease has been ascribed to the B-cell lymphoma 2 (Bcl-2) family of proteins. These proteins regulate the permeability of the outer mitochondrial membrane and thereby control cell survival, morphology, dynamics, and membrane potential of mitochondria. Bcl-2 family members can be both prosurvival and proapoptotic. The main family members inhibiting cell death are Bcl-2 and B-cell lymphoma-extra large (Bcl-xL) while the BH3-only proteins Bax and Bcl-2 antagonist (Bak) form pores in the mitochondrial membrane and thus initiate apoptosis. For SCA 3 and SCA 7 it was shown that the mRNA and protein levels of Bcl-xL were downregulated in cerebellar neurons when polyQ expanded ataxin-3 and ataxin-7, respectively, were overexpressed leading to activation of caspase-3 and caspase-9, two main caspases involved in mitochondrial induced apoptosis [53, 274]. Recently, it was shown that a direct interaction between ataxin-3 and Bcl-xL exists and suggested that ataxin-3 promotes the interaction between Bcl-xL and Bax [274]. SCA 3 and SCA 7* in vivo* models also showed increased levels of Bax mRNA and protein which can be explained by increased levels of active phospho-p53, a transcription factor known to enhance the transcription of Bax [53, 274–276]. Similarly, Bax levels were found to be increased in HD cell and mouse models [51, 268, 277] as well as in the caudate nucleus of HD patients compared to healthy individuals [278]. Moreover, polyQ expanded ataxin-3 was found to decrease mRNA and protein levels of the prosurvival Bcl-2 by affecting Bcl-2 mRNA stability [279, 280]. For HD, the alterations of Bcl-2 levels remain controversial. While expression of mHtt decreased Bcl-2 protein levels in different cell lines and in brain of HD mouse models [281–283], other studies did not find alterations in well studied models like R6/1 [284].

PolyQ proteins are also known to influence the transcription of multiple genes coding for important mitochondrial proteins. One example is the impairment of peroxisome proliferator-activated receptor-*γ* (PPAR-*γ*) coactivator-1*α* (PGC-1*α*) expression and function. PGC-1*α* is a transcriptional master coactivator controlling mitochondrial biogenesis, metabolism, and antioxidant defense [285–287]. Alterations in levels and activity of PGC-1*α* have been found in HD patients and mouse models [288, 289] and polymorphisms of PGC-1*α* have been described to modify the age at onset in HD patients [290]. PGC-1*α* has also been considered a potential therapeutic target by showing that PGC-1*α* levels were restored and phenotype and survival of HD mice were improved by treatment with bezafibrate, a pan-PPAR agonist [291]. While PGC-1*α* emerges as an important player in HD pathogenesis, little is known about the involvement of this master coactivator in other polyQ disorders. The question also remains: whether this mechanism is exclusive to HD or is a common feature of many polyQ diseases.

Apart from changes in mitochondrial bioenergetics and transcription of important proteins associated with mitochondrial function and cell death, alterations in shape and motility of mitochondria have been observed in HD. Both retrograde and anterograde mitochondrial transport along axons were shown to be impaired by mHtt in cultured neurons of mouse and rat models [292, 293]. While fragmented mitochondria have been reported for many HD cell models and patients over the last decades, recent studies link this observation to GTPase dynamin related protein-1 (DRP-1). DRP-1 is one of the shaping proteins which regulate mitochondrial fission and fusion. Costa et al. [294] described a higher basal activity of calcineurin which phosphorylates DRP-1 and thereby increases its activity and translocation to mitochondria thus leading to mitochondrial fragmentation in HD models. A direct interaction between mHtt and Drp-1 and an increased enzymatic activity were also shown in brain tissue of HD patients and an HD mouse model [295]. Since the balance between fission and fusion is known to be crucial for mitochondrial function and since neuronal death caused by increased mitochondrial fragmentation has been reported for other neurodegenerative disorders like AD and PD [251], it seems that a better understanding of this pathway would be insightful into understanding the mechanisms and possible therapeutic opportunities in polyglutamine diseases.

## 7. Concluding Remarks

The neurodegenerative disorders belonging to the group of polyglutamine diseases reviewed here share features such as an inverse correlation of the CAG length with age at onset, neurological features as main presentations of the disease, and an autosomal dominant mode of inheritance. The polyglutamine expansion in these unrelated proteins converges them into common pathogenic mechanisms which can result in corresponding therapeutic interventions. In this review we describe these pathways and possible points of therapeutic entry. First, it is possible to target the stability and conversion of the expanded protein by enhancing protein refolding and degradation or preventing proteolytic cleavage and creation of the toxic fragment. Another option is to decrease the ability of the protein to reach the site of toxicity by altering its ability to translocate between the nucleus and cytoplasm. Enhancing the lysosomal and proteasomal degradation and facilitating autophagic aggregate clearance are exciting current prospects for therapy. Also, modifying the pathways of aggregation remains a viable therapeutic approach as does facilitating mitochondrial health and function. Overall, the field of polyglutamine disease offers many possibilities for disease intervention (Figure 1), although no current therapy is available.

## Abbreviations

AD: Alzheimer's disease
ALS: Amyotrophic lateral sclerosis
AR: Androgen receptor
ATP: Adenosine triphosphate
Bak: Bcl-2 homologous antagonist/killer
Bax: Bcl-2-associated X protein
Bcl-2: B-cell lymphoma 2
Bcl-xL: B-cell lymphoma-extra large
CAST: Calpastatin
CDK: Cyclin-dependent kinase
CHIP: C-terminus of the HSC70-interacting protein
DRP-1: GTPase dynamin related protein-1
DRPLA: Dentatorubral-pallidoluysian atrophy
HD: Huntington's disease
HDAC: Histone deacetylase
HSP: Heat shock protein
Htt: Huntingtin
iPSC: Induced pluripotent stem cell
mHtt: Mutant huntingtin
MITOL: Mitochondrial ubiquitin ligase
MMP: Matrix metalloproteinase
MSNs: Medium spiny neurons
mTOR: Mammalian target of rapamycin
NES: Nuclear export signal
NLS: Nuclear localization signal
NMDAR: N-Methyl-D-aspartate (NDMA) receptor
PSA: Puromycin-sensitive aminopeptidase
PD: Parkinson's disease
polyQ: Polyglutamine
PGC-1*α*: Peroxisome proliferator-activated receptor-*γ* (PPAR-*γ*) coactivator-1*α*
SBMA: Spinal-bulbar muscular atrophy
SCA: Spinocerebellar ataxia
TBP: TATA-binding protein
tNAA: N-Acetylaspartate + N-acetylglutamate
UIM: Ubiquitin interacting motif
UPS: Ubiquitin-proteasome system
VPA: Valproic acid.
